# PPA1 promotes NSCLC progression via a JNK- and TP53-dependent manner

**DOI:** 10.1038/s41389-019-0162-y

**Published:** 2019-09-24

**Authors:** Dehong Luo, Daishun Liu, Wen Shi, Huimin Jiang, Wei Liu, Xiaoyuan Zhang, Yonghua Bao, Wancai Yang, Xiaojun Wang, Chaoyang Zhang, Hui Wang, Liying Yuan, Yanpei Chen, Tianyin Qu, Dong Ou, Wenzhi Shen, Shuang Yang

**Affiliations:** 1 The Third Affiliated Hospital of Zunyi Medical University/ First People’s Hospital of Zunyi, Zunyi, 563200 China; 2 Tianjin Key Laboratory of Tumor Microenvironment and Neurovascular Regulation, Medical College of Nankai University, Tianjin, 300071 China; 3 Department of Pathology and Institute of Precision Medicine, Jining Medical University, Jining, 272067 China; 4 Zunyi Medical College, Zunyi, 563006 China; 5 Department of Radiation Oncology, Quanzhou First Hospital Affiliated to Fujian Medical University, Quanzhou, 362000 China; 6 Department of Biochemical Engineering, School of Chemical Engineering & Technology, Tianjin University, Tianjin, 300072 China

**Keywords:** Lung cancer, Molecular biology

## Abstract

Inorganic pyrophosphatase (PPA1) promotes tumor progression in several tumor types. However, the underlying mechanism remains elusive. Here, we disclosed that PPA1 expression is markedly upregulated in lung carcinoma tissue versus normal lung tissue. We also found that the non-small cell lung cancer (NSCLC) cell lines show increased PPA1 expression levels versus normal lung cell line control. Moreover, the knockdown of PPA1 promotes cell apoptosis and inhibits cell proliferation. Whereas, the ectopic expression of PPA1 reduces cell apoptosis and enhances cell proliferation. Most interestingly, the expression of mutant PPA1 (D117A) significantly abolishes PPA1-mediated effect on cell apoptosis and proliferation. The underlying mechanism demonstrated that TP53 expression deficiency or JNK inhibitor treatment could abolish PPA1-mediated NSCLC progression. In summary, the aforementioned findings in this study suggest a new pathway the PPA1 mediates NSCLC progression either via TP53 or JNK. Most important, the pyrophosphatase activity is indispensible for PPA1-mediated NSCLC progression. This may provide a promising target for NSCLC therapy.

## Introduction

Non-small cell lung cancer (NSCLC) is the most common tumor type (~85%) in lung cancer which has a high mortality in the worldwide^1^. As typically diagnosed at a distant stage, the 5-year survival rate of NSCLC patients are less than 15%^2^. Although recent advances in multimodality therapy improve the clinical outcome^3^, patients with NSCLC still have a high rate of relapse and will die because of cancer recurrence^4,5^. Therefore, it is urgent to develop novel molecular prognosis biomarkers for predicting the prognosis and identifying the high-risk subgroup of NSCLC patients with early stage who might benefit from comprehensive therapy.

PPA1 (inorganic pyrophosphatase) was a soluble cytosolic pyrophosphatase, it was found to play an essential role for growth and development in the Caenorhabditis elegans and round-worms Ascaris^6,7^. In the mammals, PPA1 has been demonstrated to regulate neurite growth via a JNK dephosphorylation manner in mouse neuroblastoma cells^8^, as well as it was proved to induce type I collagen synthesis and stimulate calcification by osteoblasts^9^. Also researches indicated that PPA1 expression and activity increasing in rat and mouse livers are correlated with aging^10,11^.

PPA1 was reported to be increased in various types of neoplasms like breast cancer^12^, colorectal cancer^13^, hepatocellular carcinoma^14^, lung adenocarcinoma^15^, prostate cancer^16^, and ovarian cancer^17,18^. In our previous work, we also found that the expression of PPA1 in the 12 tumor types (contained lymphadenoma, thyroid cancer, brain tumors, breast cancer, soft tissue tumors, hepatocellular carcinoma, lung cancer, ovarian cancer, prostate cancer, bladder cancer, colorectal cancer, and stomach cancer) is different and is significantly higher in lung and ovarian cancer, and its expression in lung cancer was correlated with age, smoking status, and tumor size^19^. However, the specific role of PPA1 in lung cancer or NSCLC progression remains unclear.

Here, we found that PPA1 was upregulated in lung carcinoma versus normal tissue, and its expression was correlated with patient survival. Knockdown of PPA1 promoted cell apoptosis and inhibited cell proliferation in NSCLC cells. The ectopic expression of PPA1 enhanced cell proliferation ability and reduced cell apoptosis, however, the expression of mutant PPA1 (D117A) attenuated PPA1-mediated regulation of cell proliferation and cell apoptosis. TP53 is a famous tumor suppressor gene which could triggers cell apoptosis and inhibit cell proliferation. Exploration of the underlying mechanism of PPA1 demonstrated that TP53 expression and JNK activation were both indispensable for PPA1 mediated NSCLC progression. Our research may reveal a promising target for NSCLC therapy.

## Results

### The expression of PPA1 in lung carcinoma patients

**Fig. 1:**
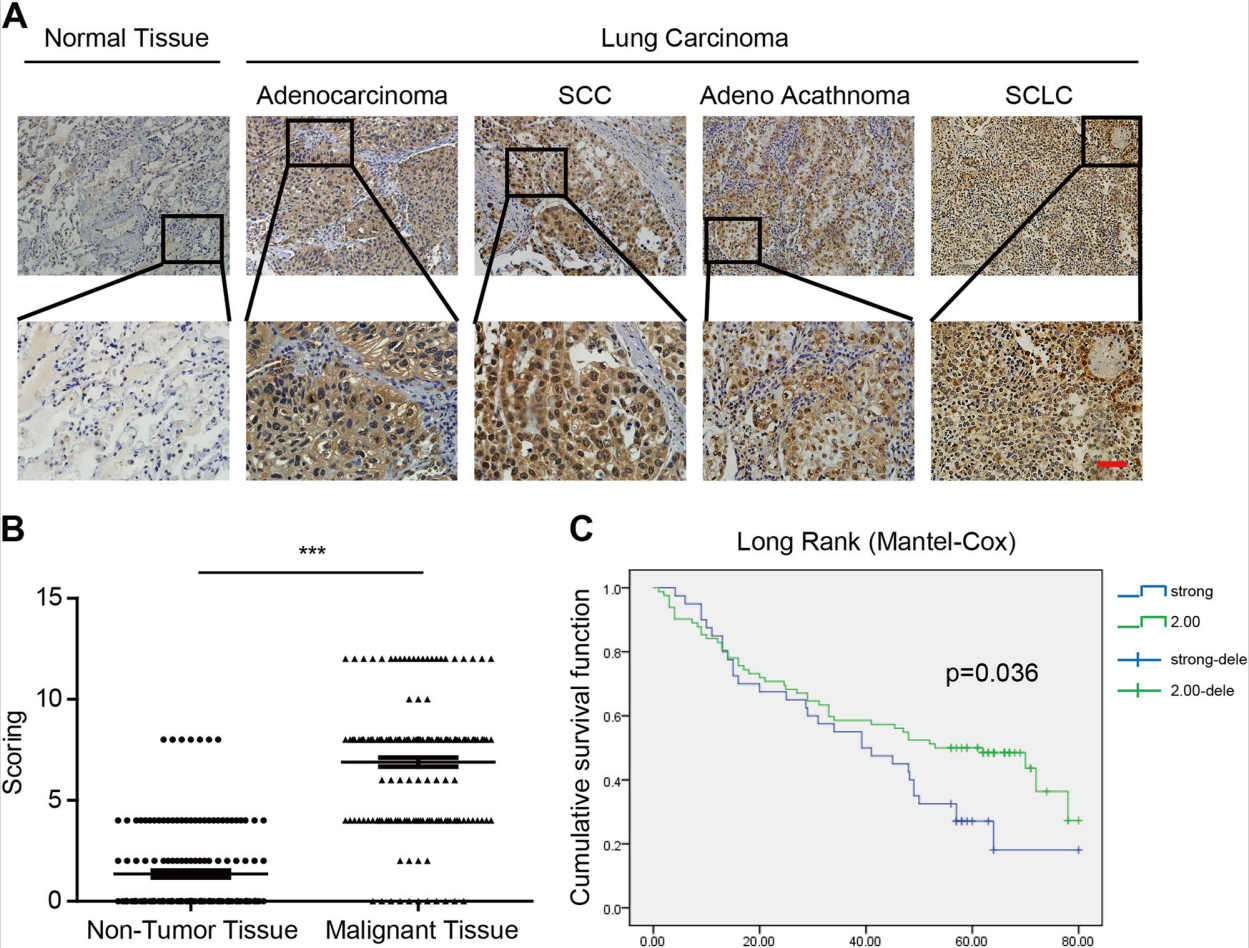
PPA1 had a high expression in patient tissues with lung cancer. **a** Immunohistochemical staining for PPA1 in paraffin-embedded samples of lung cancer and its corresponding non-cancer tissue samples. The expression of PPA1, which appeared as distinct brown staining located in the cytoplasm of positive cells, was recorded under a ×20 objective and is shown separately as representations of malignant tumors and non-tumor tissue. Scale bar = 50 µm. **b** The correlation between PPA1 expression and tumor malignancy was analyzed in lung cancer. The expression levels are presented as filled triangles and were compared using an unpaired Student’s *t*-test. **c** Univariate analysis relationship between PPA1 expression and survival in patients with lung cancer.

To explore the clinical significance of PPA1 in lung carcinoma, we performed the immunohistochemical analysis to detect the expression of PPA1 with a PPA1-specific antibody in frozen primary lung carcinoma samples (*n* = 185, contained 62 adenocarcinoma, 93 SCC, 9 adeno acathnoma and 21 SCLC) and normal lung tissues (*n* = 10) (Fig. 1a). The results showed that a relatively high expression of PPA1 was detectable in lung tumor tissues versus normal tissue (Fig. 1b). We also performed the bioinformatic analysis (univariate analysis) to disclose the correlation between PPA1 expression and patient survival, the results showed that higher expression of PPA1 correlates with poor survival of the patients (Fig. 1c). Together, these results indicated that PPA1 was upregulated in lung carcinoma tissues.

### Suppression of PPA1 promotes NSCLC cell apoptosis in vitro

**Fig. 2:**
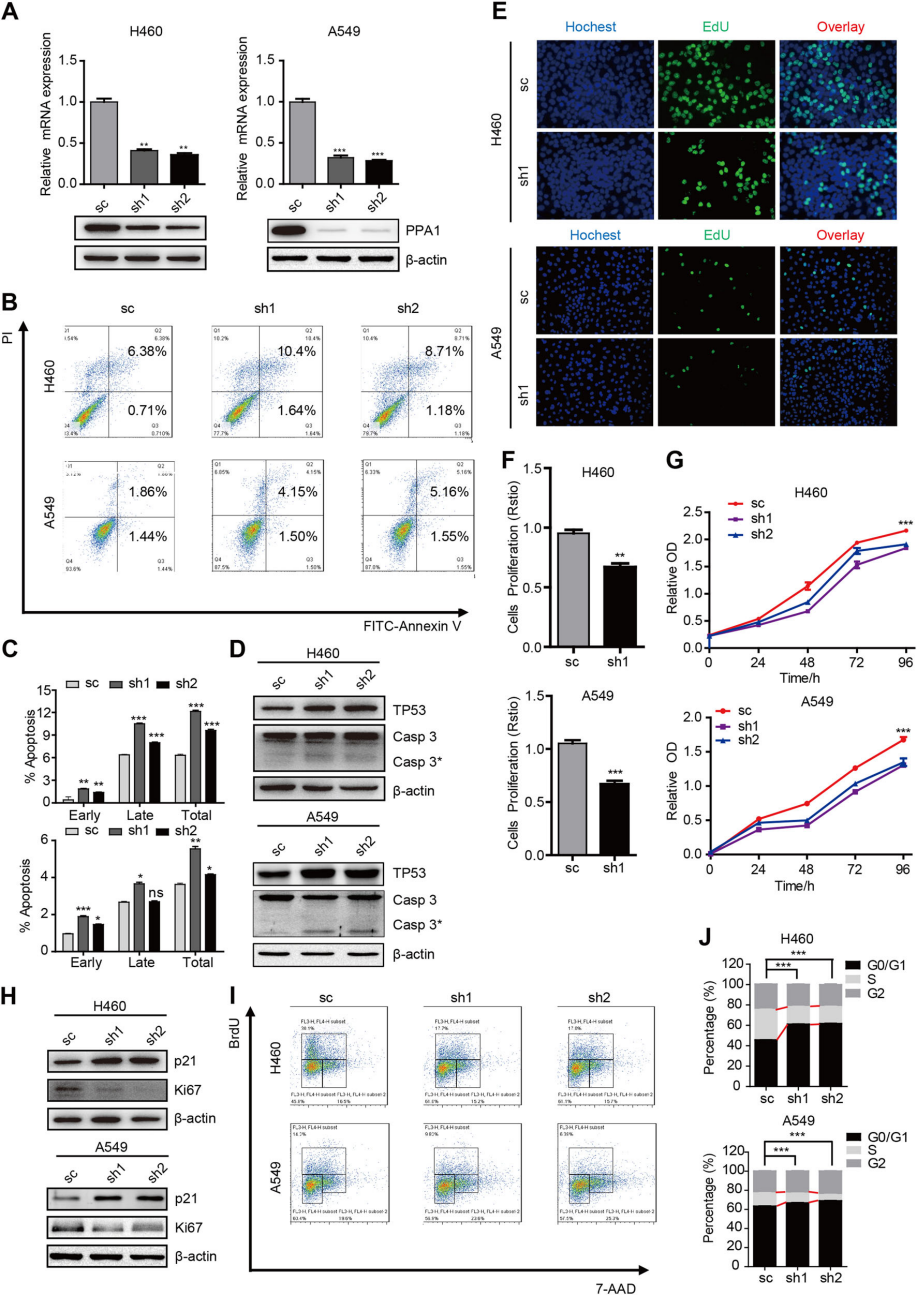
PPA1 deficiency fosters NCSLC cell apoptosis in vitro. **a** The Real-Time PCR and Western Blot results showed the efficiency of PPA1 knockdown in NCI-H460 and A549 PPA1 transduced cell lines, β-actin was included as a loading control. **b** Flow cytometry assay was performed on shCtrl and shPPA1 of NCI-H460 and A549 cells. A representative of three experiments was shown. **c** The statistical results of the apoptotic cells percentage were shown. *P < 0.05 indicate statistically significant differences versus the control group. **d** Western blot shows the relative PPA1 expression as well as the expression of P53 and cleaved caspase-3 in NCI-H460 and A549 cell lines with PPA1 knockdown, β-actin was included as a loading control. **e** EdU incorporation assays performed on shCtrl and shPPA1 of NCI-H460 and A549 cells. **f** The statistical results of EDU assay were shown. **g** The cell growth of shCtrl and shPPA1 these cells were analyzed at 0, 24, 48, 72, and 96 h after cell seeding. **h** The immunoblot showed the expression of p21 and Ki67 in shCtrl and shPPA1 NCI-H460 and A549 cell lines with PPA1 knockdown. **i** BrdU-7AAD double staining assay was performed on NCI-H460 and A549 with silencing PPA1. **j** The statistical results of S stage percentage in NCI-H460 and A549 with silencing PPA1or shCtrl were shown.

In our previous work, we found that PPA1 was also increased in human NSCLC cell lines (H460, H1299, and A549) compared to normal control MRC-5^19^. To investigate the functional role of PPA1 in tumor progression, we knocked down PPA1 expression in H460 and A549 cell lines, as shown in Fig. 2a, the mRNA and protein level both decreased by using PPA1 specific shRNAs. PI-Annexin V double-staining assay was initially performed to detect the function of PPA1 interference in the cell apoptosis. As shown in Fig. 2b, in H460 cells, knockdown of PPA1 expression promoted cell apoptosis ability of both cell lines (Fig. 2c). Western blot assay was also performed to check the expression of TP53 and the cleaved-caspase3, and the results showed that knockdown PPA1 increased the expression of both TP53 and the cleaved-caspase3 (Fig. 2d). These observations showed an indicative role of PPA1 in inhibiting NSCLC cell apoptosis.

### PPA1 deficiency suppresses NSCLC cell proliferation in vitro

As proliferation was one of the key elements of tumor progression, next we investigated the function of PPA1 in NSCLC cell proliferation. We performed the EdU fluorescence staining assay to test the cell proliferation ability in H460 and A549 cell lines (Fig. 2e). We found that knockdown of PPA1 expression decreased the cell proliferation ability (Fig. 2f). We also used the CCK-8 assay to detect the cell viability and the results showed that knockdown of PPA1 expression inhibited the speed of cell growth in the indicated time points (Fig. 2g). Then the expression of proliferation-related proteins was detected, showing that PPA1 deficiency increased the expression of p21 but decreased the expression of Ki-67 (Fig. 2h). Moreover, the BrdU assay was performed to analyze the cell cycle progression, the results revealed that PPA1 knockdown reduced the amounts of cells in S phase, which further suppressed the cell proliferation ability (Fig. 2i, j). Thus, these results indicated that PPA1 promotes NSCLC cell proliferation.

### PPA1 interference-induced tumor suppression is abolished in TP53-deficient H1299 cells

**Fig. 3:**
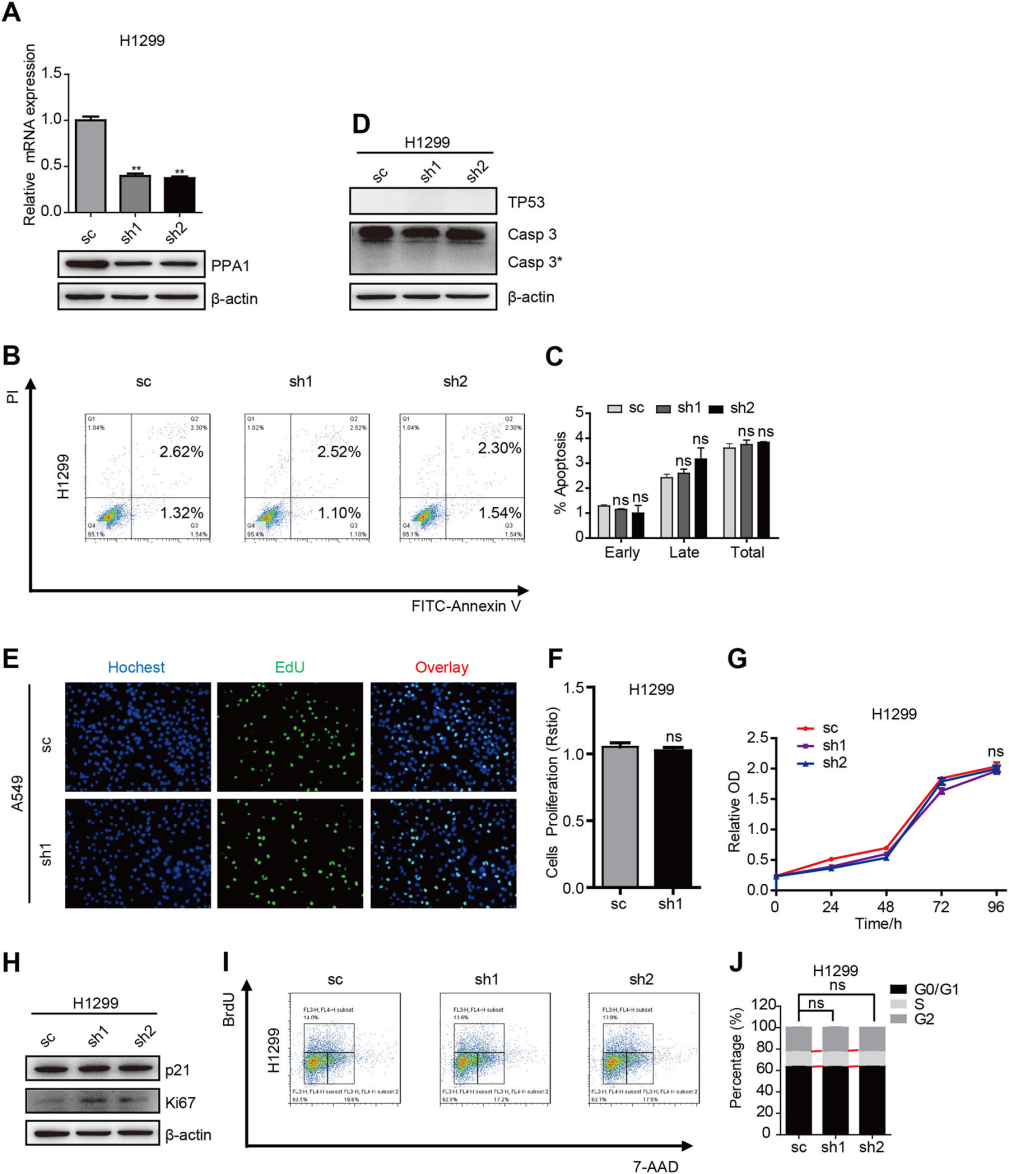
PPA1 deficient mediates tumor suppression was abolished in TP53 dificient NCI-H1299 cells. **a** The Real-Time PCR and western blot results showed the efficiency of PPA1 knockdown in NCI-H1299 PPA1 transduced cell lines, β-actin was included as a loading control. **b** Flow cytometry assay was performed on NCI-H1299 cells with silencing PPA1or shCtrl. A representative of three experiments was shown. **c** The statistical results of the percentage of apoptotic cells were also shown, which indicated no significant differences versus the control group. **d** Western blot showed the relative PPA1 expression as well as the expression of P53 and cleaved caspase-3 in NCI-H1299 cell lines with PPA1 knockdown, β-actin was included as a loading control. **e** EdU incorporation assays performed on shCtrl and shPPA1 of NCI-H1299 cells were shown. **f** The statistical results of EDU assay were shown. **g** The cell growth of shCtrl and shPPA1-NCI-H1299 were analyzed at 0, 24, 48, 72, and 96 h after cell seeding. **h** The immunoblot showed the expression of p21 and Ki67 in shCtrl and shPPA1 NCI-H1299 cell lines with PPA1 knockdown. **i** BrdU-7AAD double staining assay was performed on NCI-H1299 with silencing PPA1 or shCtrl. **j** The statistical results of S stage percentage in NCI-H1299 with silencing PPA1 or shCtrl were shown.

Our previous study showed that PPA1 is closely correlated with TP53^19^. Given that H1299 is a TP53-deficient cell line, we further verified the function of PPA1 in H1299 cells. The mRNA and protein levels were both decreased by using PPA1 specific shRNAs (Fig. 3a). PI-Annexin V double staining assay was performed to check the cell apoptosis (Fig. 3b), to our surprise, the results showed that knockdown PPA1 expression had no effects on cell apoptosis ability (Fig. 3c). In addition, western blot assay was also performed to check the expression of cleaved-caspase3, the results confirmed that knockdown of PPA1 expression had no effects on cleaved-caspase3 expression (Fig. 3d).

We also examined the effects of PPA1 on cell proliferation in TP53-deficient H1299 cells. The results of EdU fluorescence staining and CCK-8 assay revealed that knockdown of PPA1 expression had no effect on cell proliferation ability (Fig. 3e–g). Moreover, no obvious alternation of p21 and Ki-67 expression was observed (Fig. 3h). In line with this, the BrdU staining results showed that the amounts of cells in S phase also made no difference (Fig. 3i, j). Collectively, these results suggested that PPA1-mediated regulation of cell apoptosis and proliferation was mainly depending on TP53 expression.

### PPA1 reconstitution suppresses NSCLC cell apoptosis and relies on its pyrophosphatase activation in vitro

**Fig. 4:**
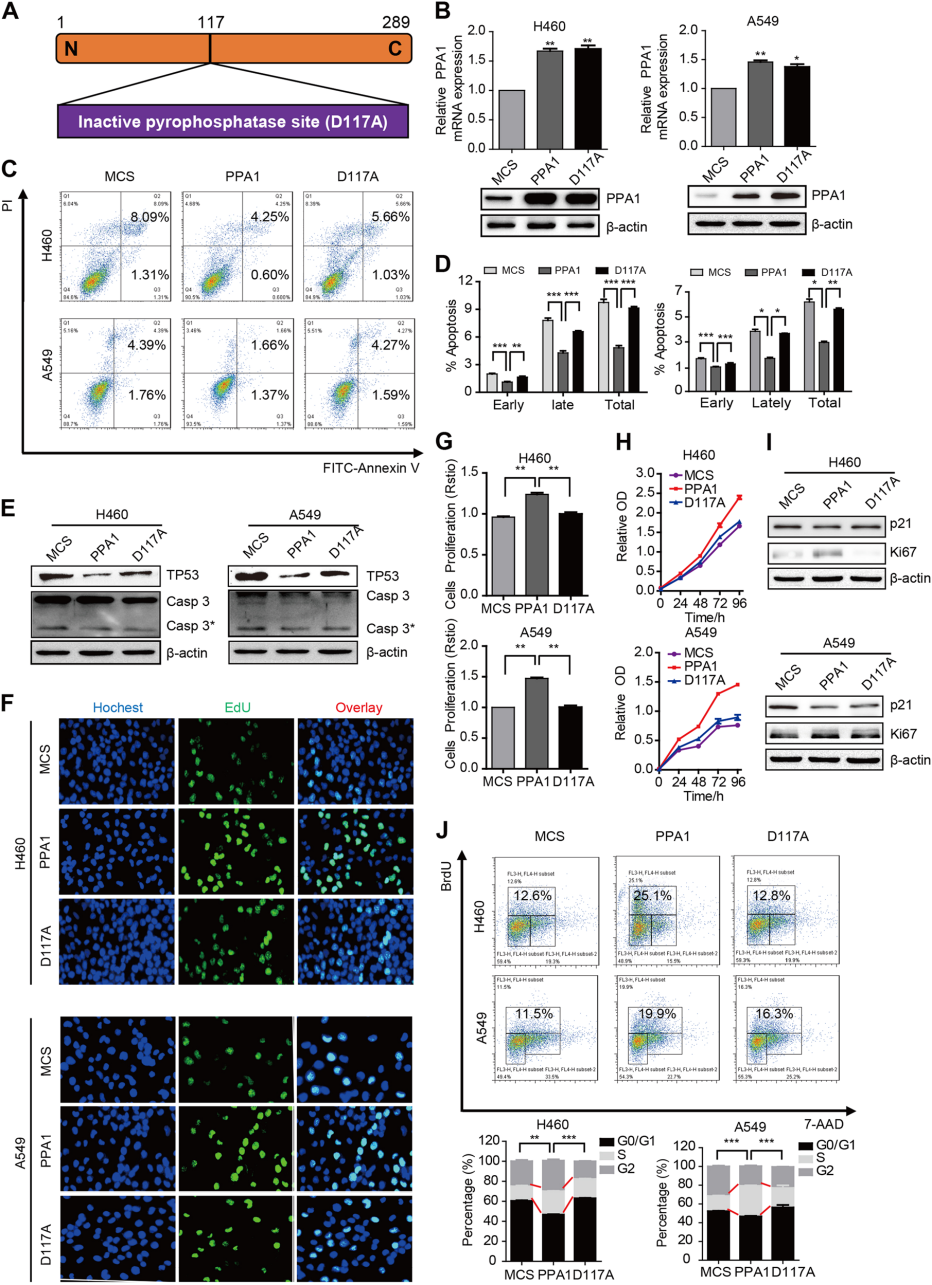
PPA1 reconstitution suppresses NSCLC cell apoptosis depend on pyrophosphatase activation in vitro. **a** Schematic structure of an inactive pyrophosphatase mutant PPA1 (D117A) was shown. **b** The over-expressing efficiency of PPA1 and D117A in NCI-H460 and A549 were detected by real-time PCR and western blot. *P < 0.05 indicate statistically significant differences versus the control group. **c** Flow cytometry assay was performed on NCI-H460 and A549 cell lines with over-expressing PPA1 and D117A. A representative of three experiments was shown. **d** The statistical results of the apoptotic cells percentage were shown, *P < 0.05 indicate statistically significant differences versus the control group. **e** Western Blot showed the relative PPA1 expression as well as the expression of TP53 and cleaved caspase-3 in the previous cell lines, β-actin was included as a loading control. **f** EdU incorporation assays performed on NCI-H460 and A549 cell lines with over-expressing PPA1 and D117A. **g** The statistical results of EDU assay were shown. *P < 0.05 indicate statistically significant differences versus the control group. **h** The cell growth of NCI-H460 and A549 cell lines with over-expressing PPA1 and D117A were analyzed at 0, 24, 48, 72, and 96 h after cell seeding. **i** The immunoblot showed the expression of P21 and Ki67 in the same cell lines with over-expressing PPA1 and D117A, β-actin was included as a loading control. **j** BrdU-7AAD double staining assay was performed on NCI-H460 and A549 with over-expressing PPA1 and D117A. The statistical results of S stage percentage in NCI-H460 and A549 with over-expressing PPA1 and D117A were also shown.

To further identify the molecular mechanism, we reconstituted either wild type PPA1 (PPA1), or an inactive pyrophosphatase-mutant PPA1 (D117A) (Fig. 4a) into NSCLC cell lines. The mRNA and protein levels were both increased in H460 and A549 cell lines (Fig. 4b). The result of PI-Annexin V double staining noticed that the cell apoptosis was decreased upon the expression of PPA1; however, expression of D117A did not inhibit cell apoptosis in both cell lines (Fig. 4c, d). Western blot assays also showed that the expression of TP53 and cleaved-caspase3 was reduced by PPA1 reconstitution, but that of D117A was not as evident (Fig. 4e). The above findings indicated that PPA1 reconstitution suppresses NSCLC cell apoptosis, which effect depended on its pyrophosphatase activation.

### PPA1 reconstitution fosters NSCLC cell proliferation and relies on its pyrophosphatase activation in vitro

To determine the effects of PPA1 and the functional mutant D117A on cell proliferation, the EdU staining assay was performed (Fig. 4f) and the results noticed that the cell proliferation ability was enhanced upon PPA1 reconstitution, and this effects was abolished by the pyrophosphatase site mutation D117A (Fig. 4g). Moreover, the CCK-8 assay results displayed that PPA1 reconstitution accelerated the cell growth at the indicated time points; however, the D117A showed no effect (Fig. 4h). Also, the expression of p21 was decreased and Ki-67 was increased following PPA1 reconstitution, but in D117A group, their expression had nearly no change (Fig. 4i). In agreement, the BrdU staining results showed that the amount of cells in S phase was increased, but in D117A group, their amount made no difference (Fig. 4j). In summary, these results disclosed that PPA1 reconstitution fosters NSCLC cell proliferation depend on pyrophosphatase activation in vitro.

### PPA1 reconstitution-mediated tumor progression depends on TP53 expression

**Fig. 5:**
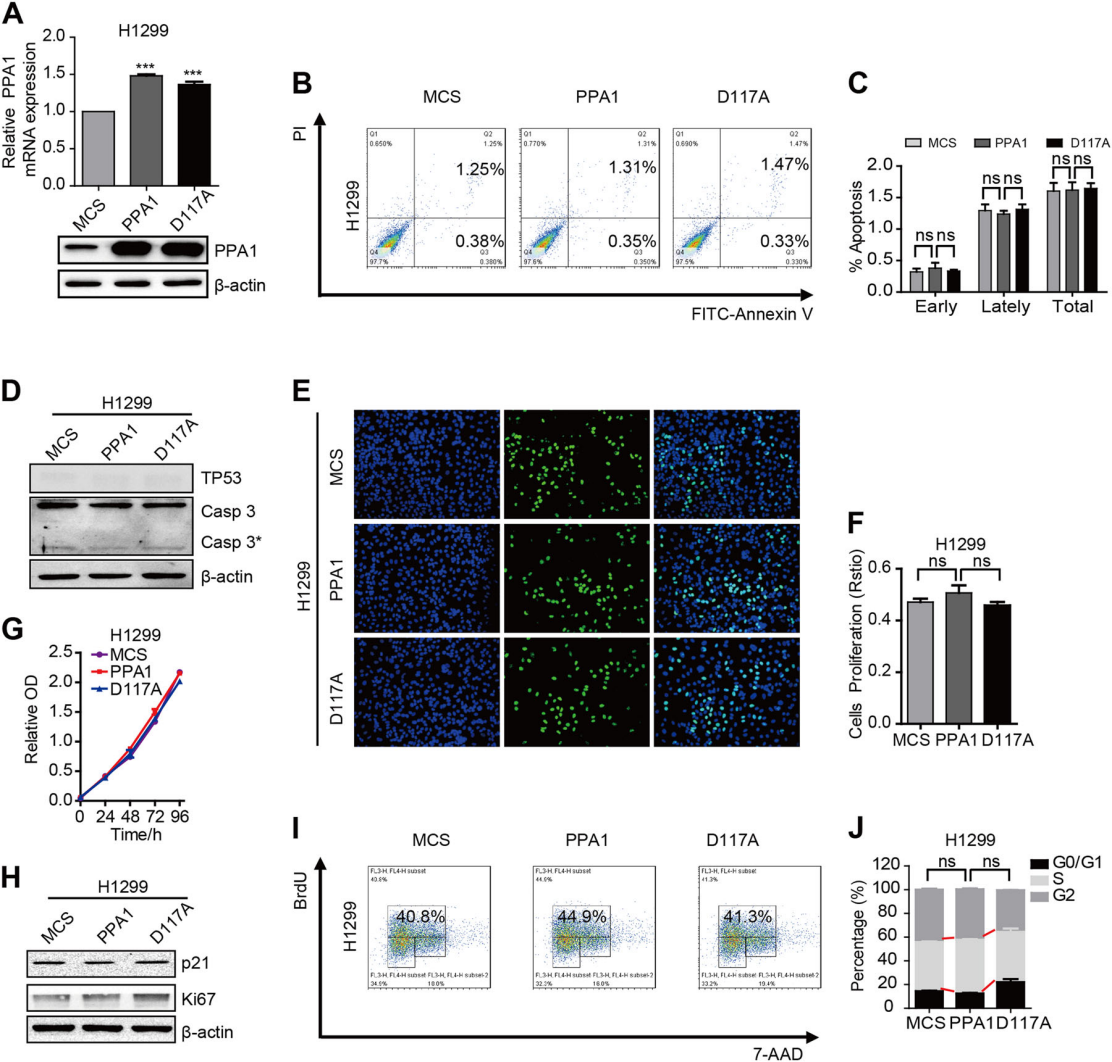
PPA1 reconstitution mediated tumor progression depends on TP53 expression. **a** The over-expressing efficiency of PPA1 and D117A in NCI-H1299 was detected by real-time PCR and western blot. **b** Flow cytometry assay was performed on NCI-H1299 cells with over-expressing PPA1 and D117A. A representative of three experiments was shown. **c** The statistical results of the apoptotic cells percentage were shown. **d** Western blot showed the relative PPA1 expression as well as the expression of TP53 and cleaved caspase-3 in H1299 cell lines, β-actin was included as a loading control. **e** EdU incorporation assays performed on NCI-H1299 cells lines. **f** The statistical results of EDU assay were shown. **g** The cell growth of NCI-H1299 cell lines with over-expressing PPA1 and D117A were analyzed at 0, 24, 48, 72, and 96 h after cell seeding. **h** The immunoblot showed the expression of P21 and Ki67 in the same cell lines with over-expressing PPA1 and D117A, β-actin was included as a loading control. **i** BrdU-7AAD double staining assay was performed on NCI-H1299 with over-expressing PPA1 and D117A. **j** The statistical results of S stage percentage in NCI-H1299 with over-expressing PPA1 and D117A were also shown.

As PPA1-regulated tumor suppression was depended on TP53 expression, we also reconstituted either PPA1 or D117A into TP53-deficient H1299 cell lines. The mRNA and protein levels were both increased in H1299 cell lines (Fig. 5a). PI-Annexin V double-staining results showed that the cell apoptosis showed no alteration following reconstitution of either PPA1 or D117A (Fig. 5b, c). Western blot also confirmed that the expression of cleaved-caspase3 was not regulated by ectopic PPA1 and D117A (Fig. 5d).

We also examined the effects of PPA1 on cell proliferation in TP53-deficient H1299 cell lines. EdU fluorescence staining results revealed that PPA1 or D117A reconstitution made no difference on cell proliferation ability (Fig. 5e, f). Moreover, the CCK-8 assay results displayed that PPA1 or or D117A reconstitution had no significant effects on cell growth in several time points (Fig. 5g). Also, the expression of p21 and Ki-67 had no change (Fig. 5h). In addition, the BrdU staining results showed that the amount of cells in S phase also made no difference with the PPA1 or D117A reconstitution (Fig. 5i, j). Taken together, these results indicated that TP53 expression was indispensible for PPA1 reconstitution-mediated tumor progression.

### PPA1 restricts JNK activation to mediate NSCLC tumor progression in vitro

**Fig. 6:**
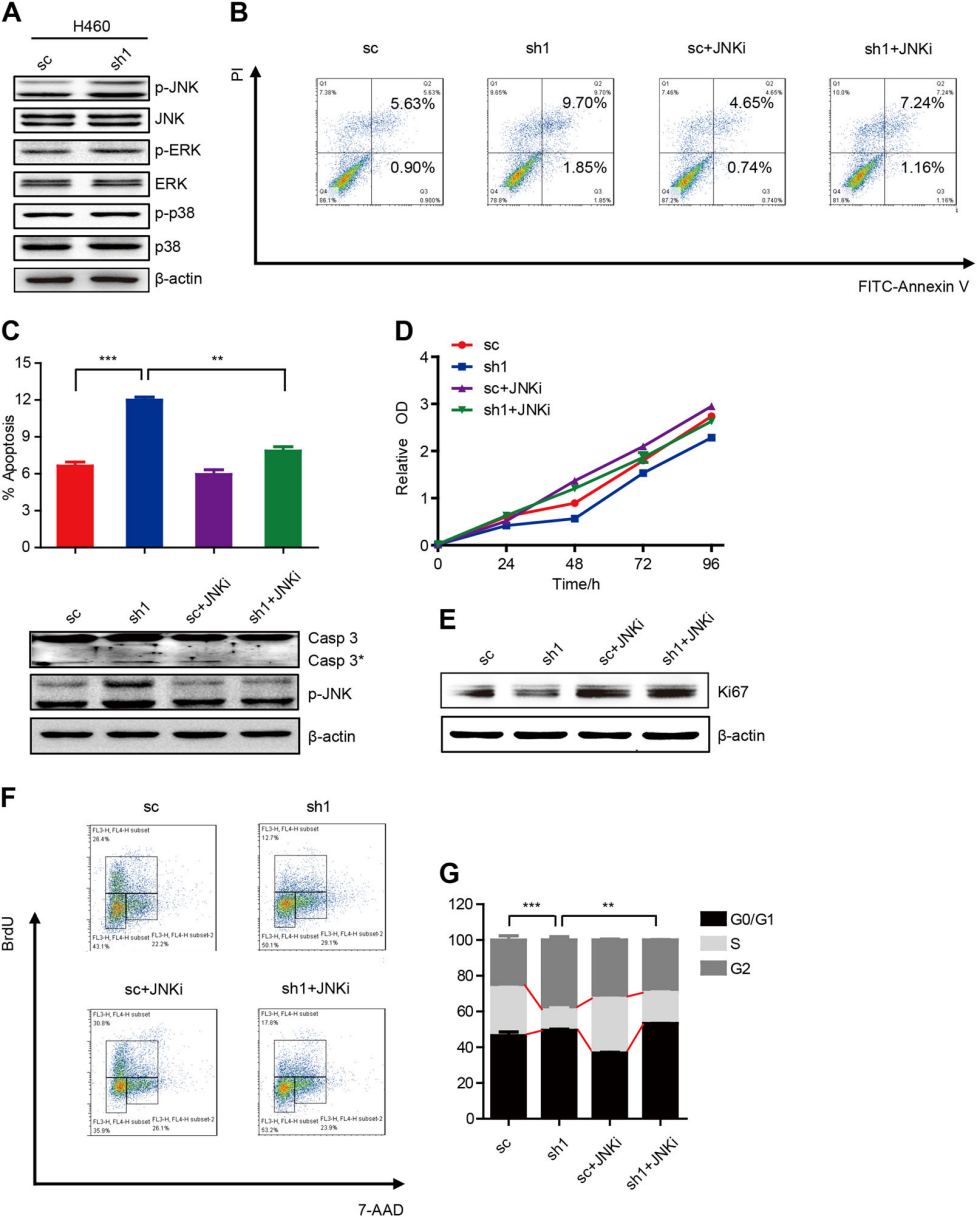
PPA1 restricts JNK activation to mediate NSCLC tumor progression in vitro. **a** Western Blot was used to detect the expression of JNK, ERK, p38, and their activation form, β-actin was included as a loading control. **b** Flow cytometry assay was performed on NCI-H460 silencing PPA1 with or without adding JNK420119. **c** Up panel: the statistical result of the flow cytometry analysis. Asterisks represent *p*-values < 0.05. Down panel: western blot was used to detect the expression of P-JNK and Caspase3 in NCI-H460 silencing PPA1 with or without adding JNK420119. **d** The cell growth of NCI-H460 with silencing PPA1 after added or not added JNK420119 were analyzed at 0, 24, 48, 72, and 96 h after cell seeding, NCI-H460 SC as negative control. **e** The immunoblot showed the expression of Ki67 in NCI-H460 silencing PPA1 with or without adding JNK420119, β-actin was included as a loading control. **f** BrdU-7AAD double staining assay was performed on NCI-H460 silencing PPA1 with or without adding JNK420119. **g** The statistical results of S stage percentage in NCI-H1299 silencing PPA1 with or without adding JNK420119.

The previous report proved that PPA1 could directly dephosphorylate pJNK1^8,20,21^. To investigate this role of PPA1 in NSCLC tumor progression, we first checked the phosphorylation of JNK in PPA1-interferd H460 cells. Western blot showed that JNK phosphorylation was increased upon knockdown of PPA1 expression; however, the phosphorylation of ERK and p38 was not as evident (Fig. 6a). Then we used the specific inhibitor for JNK to suppress its phosphorylation and activation and detected the cell apoptosis (Fig. 6b). PI-Annexin V double-staining results showed that knockdown of PPA1 expression promoted cell apoptosis while JNK inhibitor could abolish this effect restored cell apoptosis ability (Fig. 6c, up). The same observation was obtained by the expression of cleaved-caspase3 (Fig. 6c, down).

We also evaluated the function of JNK activation in PPA1-mediated NSCLC cell proliferation. The CCK8 assay results displayed that knockdown of PPA1 expression inhibited cell apoptosis while JNK inhibitor could also recover cell proliferation ability to normal (Fig. 6d). The same results were proved by the expression of Ki-67 (Fig. 6e). Furthermore, the BrdU staining assay showed that knockdown of PPA1 resulted in decreased amounts of cells in S phase, while JNK inhibitor rescued this effect (Fig. 6f, g). Altogether, these findings suggested that JNK activation was responsible for PPA1-mediated NSCLC tumor progression in vitro.

### PPA1 fosters NSCLC tumor progression in vivo

**Fig. 7:**
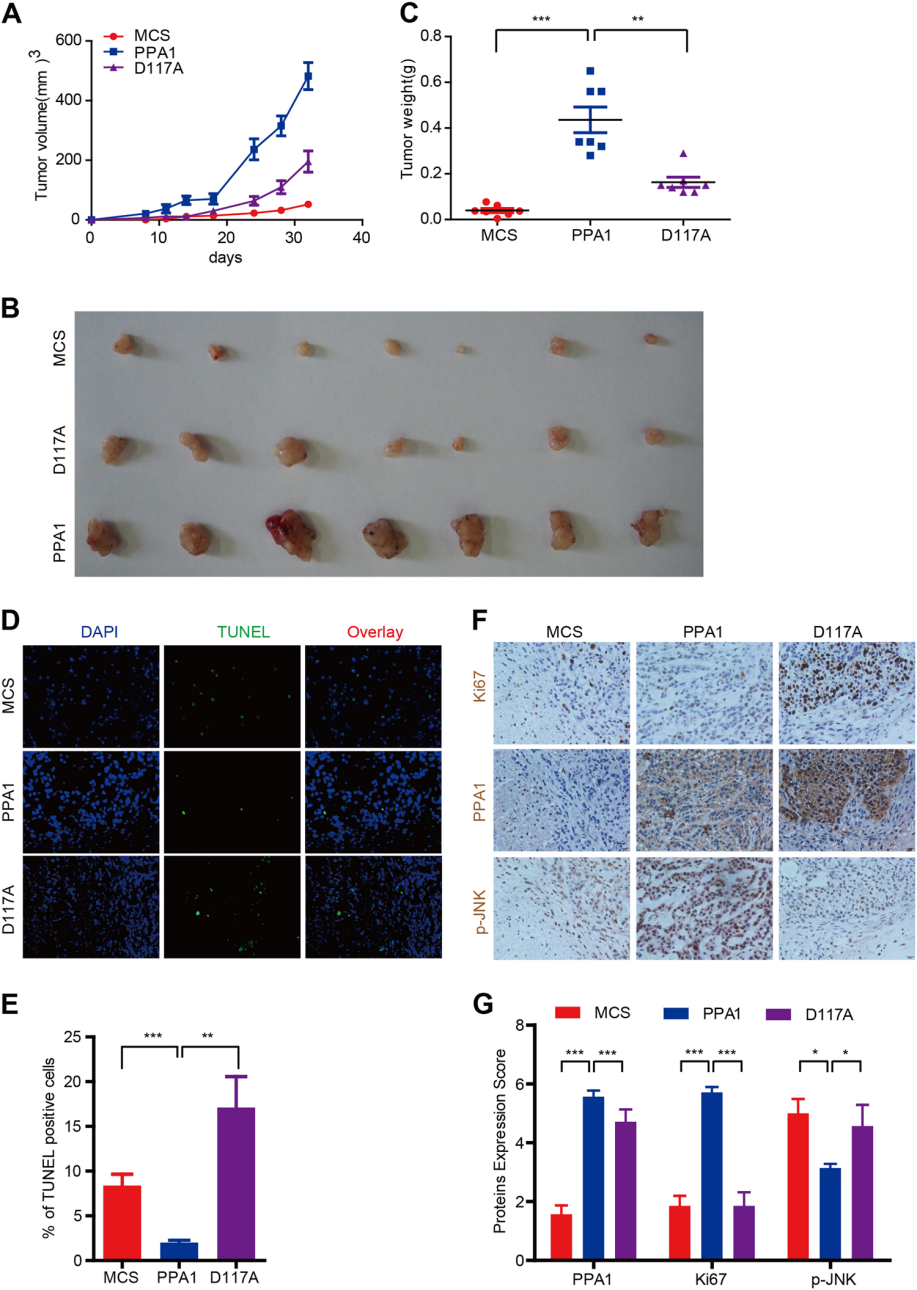
PPA1 promotes the proliferation of NSCLC tumor cells in vivo. **a** Stable A549-MCS, A549-PPA1, and A549-D117A cells were injected into the forth fat pad of NOD/SCID mice, the tumor growth curve was measured. **b** The tumors from different group mice were shown. **c** The statistical results of tumor weight were shown. **d** TUNEL staining assay was performed to detect the cell apoptosis in various group mice tumors. **e** The statistical results of the apoptotic cells (TUNEL positive) in various group mice tumors were shown. **f** IHC staining assay was performed to detect the expression of PPA1, Ki-67, and p-JNK in various group mice tumors. **g** The statistical results of the PPA1, Ki-67, and p-JNK positive cells in various group mice tumors were shown.

Since PPA1 contributes to the NSCLC tumor progression via promoting cell proliferation and inhibiting cell apoptosis in vitro, we next tested the results in a xenograft animal model. To this end, stable A549-MCS, A549-PPA1, and A549-D117A cells were injected into the forth fat pad of NOD/SCID mice, respectively. As shown in (Fig. 7a–c) the tumor growth, tumor volume and tumor weight in the A549-PPA1 group showed a marked increase versus the A549-MCS control; however, a significant reduction of the tumor growth and tumor volume was observed in A549-D117A versus the A549-PPA1 group. Moreover, the TUNEL staining results showed that the apoptotic cells (TUNEL positive) in A549-PPA1 group was apparently more than A549-MCS and A549-PPA1 group (Fig. 7d, e). In addition, consistent with the in vitro findings, the IHC staining results also revealed that PPA1 reconstitution increased the expression of Ki-67 and inhibited JNK activation, while D117A had no effects (Fig. 7f, g). These findings collectively suggested that PPA1 fosters NSCLC tumor progression in vivo.

## Discussion

Lung cancer is still the leading cause of cancer-related death in American^1^ and is the leading cause of cancer-related death worldwide, in which non-small cell lung cancer (NSCLC) accounts for ~85%^2^. Therefore, developing novel molecular prognosis biomarkers for predicting the prognosis and identifying the high-risk subgroup of NSCLC patients at early stage who might benefit from comprehensive therapy is urgently needed.

PPA1 was upregulated in multiple cancers^12,14–16,18^. The previous study in our lab^22^ found that the expression of PPA1 was significantly higher in lung cancer, and its expression was associated with age, smoking status, and tumor size, which suggests that PPA1 plays an important role in lung tumorigenesis and progression. Thus, our present study was designed to evaluate the function and potential mechanism of PPA1 in regulating NSCLC cell apoptosis and proliferation. We disclosed that knockdown of PPA1 expression promotes cell apoptosis and simultaneously inhibits cell proliferation. In contrast, PPA1 reconstitution performed the opposite effect that suppresses cell apoptosis and promotes cell proliferation. These results indicate the oncogenic property of PPA1 in NSCLC tumorigenesis and progression.

In addition, we found that TP53 expression deficiency or JNK inhibition abolished PPA1-mediated NSCLC progression. TP53 is a famous tumor suppressor gene that has been called the “cellular gatekeeper” and “guardian of the genome”. TP53 expression is closely relevant to tumor apoptosis and proliferation^23,24^. The study about PPA1 and TP53 correlation is insufficient, and the results are controversy. PPA1 silence decreased TP53 expression in diffuse large B-cell lymphoma^25^, whereas, PPA1 reconstitution decreased TP53 expression in ovarian cancer cell lines^26^. Our previous work showed that the function of PPA1 was intimately correlated with TP53 expression^19^. Here we showed that PPA1 negatively regulated TP53 expression in NSCLC and TP53 deficiency abolished the role of PPA1 in NSCLC progression, indicating that the expression of TP53 is indispensible to PPA1-mediated NSCLC tumor progression.

The role of JNK protein in cancer is contradictive. On one hand, mild activation of JNK promotes cell proliferation and invasion predominantly via c-Jun and ATF signaling pathways^27^. On the other hand, strong JNK activation phosphorylates other substrates such as p53 and Bcl-2, therefore acting as a tumor suppressor^28,29^. Previous reports proved that PPA1 activates JNK via de-phosphorylation, which regulates the proliferation of mouse neuroblastomas^8^ and chick cerebellar neurons^21^. Moreover, PPA1 protein purification and enzymatic studies proved that PPA1 specifically and directly dephosphorylate p-JNK at both phosphor-peptide and phosphor-protein levels, while no catalytic activity towards p-ERK or p-p38 was detected^20^. In our present study, we further revealed that PPA1 promotes JNK dephosphorylation, eventually leading to NSCLC tumor progression, indicating the tumor suppressor role of JNK in PPA1-mediated NSCLC progression.

Although we discovered TP53 expression or JNK activation is crucial for PPA1-mediated NSCLC progression, we did not investigate the regulation of JNK on TP53 in our present study. We will explore whether p53 is the downstream target of JNK in PPA1-mediated NSCLC progression in our future study.

In conclusion, our present study demonstrated that the expression of PPA1 was markedly upregulated in lung carcinoma tissue versus normal lung tissue. PPA1 promotes NSCLC progression via enhancing cell proliferation and reducing cell apoptosis. TP53 silencing or JNK inactivation can inhibit PPA1-mediated NSCLC progression. This may provide a promising target for NSCLC therapy.

## Materials and methods

### Patients and tissue samples

A total of 185 carcinoma tissue samples were obtained from patients who underwent surgical treatment at the TAHZMU and Medical College of Nankai University from January to December 2014. None of the patients received therapy before surgery. The tissues from all of the patients were staged according to the American Joint Committee on Cancer TNM staging system. This study was approved by the institutional ethics committees at the Third Affiliated Hospital of Zunyi Medical University.

### Immunohistochemistry

Immunohistochemistry (IHC) was performed on paraffin-embedded specimens and tissue microarrays of human lung cancer. The PPA1 expression in the tissue samples were detected by using PPA1 specific antibody (Sigma-Aldrich, St Louis, MO, USA) and scored according to the percentage of PPA1-positive cells in each lung tissues. The Ki-67 and p-JNK expression in the mice tumor tissue sections were also detected by using the Ki-67 (Abcam Biotechnology, Inc., Abcam, Hong Kong) and p-JNK (Santa Cruz Biotechnology Inc, Santa Cruz, CA, USA) specific antibodies. The images were recorded by Olympus BX51 Epi-fluorescent microscopy under a ×10 or ×40 objective (Olympus Co., Tokyo, Japan)^30^.

### Vector construction

The sequences of human PPA1 shRNA and scrambled control were summarized in Supplemental Table 1. The palindromic DNA oligos were annealed to each other to form a double-stranded oligo, which was then ligated to the linearized vector pLV-H1-EF1α-puro (cat. #SORT-B19, Biosettia Inc) to generate circled pLV-H1-shRNA-PPA1-EF1α-Puro or pLV-H1-sc-EF1α-Puro^31^.

### Cell culture

Human lung cancer cell lines (NCI-H460, A549, and NCI-H1299) were purchased from American Type Culture Collection (ATCC). All the cell lines were recently authenticated by cellular morphology and the short tandem repeat analysis at Microread Inc. (Beijing, China; May 2014) according to the guideline from ATCC. NCI-H460-WT, A549-WT, and NCI-H1299-WT cells were infected with a lenti-virus carrying the pLV-EF1α-Flag-PPA1-IRES-Bsd and pLV-EF1α-Flag-D117A-IRES-Bsd plasmids, respectively, followed by clonal selection using blasticidin to generate polyclon stable overexpression of Flag-PPA1 and D117A (NCI-H460-Flag-PPA1, NCI-H460-Flag-D117A, A549- Flag- PPA1, A549-Flag-D117A, NCI-H1299-Flag-PPA1, and NCI-H1299-Flag-D117A). Meanwhile, the aforementioned wild type cells were infected with lenti-virus carrying pLV-EF1α-MCS-IRES-Bsd plasmid used as the control^32^.

### RNA preparation and real-time PCR analysis

Total RNAs of cells were extracted by TRIzol reagent (Cat. #15596-018, Invitrogen Inc, Carlsbad, CA) and then reverse transcribed into cDNAs. Real time PCR was performed in 20 µl reaction volumes by using TransStart Green qPCR SuperMix Kit (TransGen Biotech, Beijing, China, PR). The 2-ΔΔCt method was used to determine the relative mRNA folding changes. Statistical results were averaged from three independent experiments performed in triplicate. The specific primer sequences for human PPA1 and GAPDH were summarized in Supplemental Table 1^33^.

### Western blot

Cell lysates from different cell lines were prepared with RIPA buffer in the presence of protease inhibitor cocktails and Phosphatase Inhibitor Cocktail 2 and 3 (P8340, P5726, and P0044, Sigma-Aldrich, St Louis, MO, USA). Primary antibodies included the following: anti-Ki67 (Abcam Biotechnology, Inc., Abcam, Hong Kong), anti-P21, anti-caspase3, anti-P53, anti-p-JNK, anti-JNK, anti-p-P38, anti-P38, anti-p-ERK, anti-P38, and anti-β-actin (Santa Cruz Biotechnology Inc, Santa Cruz, CA, USA), and anti-PPA1 (Sigma-Aldrich St Louis, MO, USA)^34^.

### 5-ethynyl-2’-deoxyuridine (EdU) cell proliferation assay

In total, 1 × 10^5^ of polyclone stable cells were seeded in a 24-well plate and incubated for 24 h. Then, an EdU (5’-ethynyl-2’-deoxyuridine) incorporation assay was performed to quantify cell proliferation using a Cell-Light™ EdU DNA Cell Proliferation Kit (Guangzhou Ribobio Co., Ltd, Guangzhou, China) according to the manufacturer’s instructions. Cell groups that were not subsequently treated with EdU served as the negative controls.

### Cell proliferation assay

Cell proliferation was quantified by the CCK-8 cell proliferation assay, which generated a growth curve from 0 to 96 h. Cells were seeded at a density of 2–5 × 10^3^ cells per well in a 96-well plate (day 0) and incubated for an additional 4 days. At the time of collection, 10 µl of the kit reagent was added to each well, and the cells were allowed to incubate for 2 h at 37 °C. Thereafter, the optical density (OD) was measured at 450 nm using a 96-well multiscanner autoreader (Thermo Electron Corp, Waltham, MA, USA).

### The cell cycle and apoptosis assay

Cells were cultured in the absence of fetal bovine serum for 12 h, then treated with 10 mM 5-bromo-2-deoxyuridine for 24 h, and cell cycle assay was performed by using the Cytofix/Cytoperm kit (BD Biosciences, San Jose, CA, USA) following the manufacturer’s instructions. For apoptosis assay, apoptotic cells were stained with propidium iodide and Annexin V-FITC (BD Biosciences). Flow cytometry analysis was performed by FACS Calibur cytometer (BD Biosciences), in which a minimum of 10,000 events were recorded. Three independent assays were conducted for these experiments, and the mean values were expressed as the mean ± the standard deviation (s.d.).

### TUNEL staining

Paraffin-embedded tissue slides were prepared from the tumor xenografts DeadEnd Fluorometric TUNEL System kit (Promega, Madison, WI, USA) was applied for TUNEL staining. Experiment procedure was performed according to the manuscript instruction. 4,6-Diamidino-2-phenylindole was used to stain the nuclei, and the tissue slides were subjected to Olympus BX51 Epi-fluorescent microscopy under a ×40 objective (FV1000-IX81, Olympus Microsystems, Shanghai, China).

### Inhibitors treatment

NCI-H460-shPPA1 and NCI-H460-sc cells were seeded at a density of 0.5 × 10^6^ per 60 mm dish. 24 hours after seeding, the cells were treated with 1 μM JNK420119 (JNKi) for 48 h and dimethyl sulfoxide used as the Ctrl, then cells were harvested for FACS, CCK8 or protein extraction.

### Tumor xenografts

With stronger and larger bodies, the male NOD/SCID mice at 6–8 weeks of age were chose and separated randomly into three groups (*n* = 10). In all, 3 × 10^6^ A549 cells (A549 -MCS, A549-PPA1, and A549-PPA1-D117A) were inoculated subcutaneously into each mouse at right axilla, respectively. Tumor volume (mm^3^) was measured with calipers two times per week and calculated by using the standard formula: length × width^2^/2. The individual measuring the mice was unaware of the identity of the group measured. Animal use complied with Nankai University Animal Welfare Guidelines.

### Statistical analyses

Statistical analyses were performed using SPSS 13.0 software. The values are expressed as the mean ± s.d. The significance of the differences in PPA1 expression among lung cancer and the corresponding normal specimens was established by Fisher’s exact test. The relationship between PPA1 expression and clinicopathological factors was examined by the Pearson χ^2^ method. Two-sided *P*-values < 0.05 were considered statistically significant^35^.

## Supplementary information

Supplemental tables
