# Correction: PPA1 promotes NSCLC progression via a JNK- and TP53-dependent manner

**DOI:** 10.1038/s41389-019-0184-5

**Published:** 2019-12-13

**Authors:** Dehong Luo, Daishun Liu, Wen Shi, Huimin Jiang, Wei Liu, Xiaoyuan Zhang, Yonghua Bao, Wancai Yang, Xiaojun Wang, Chaoyang Zhang, Hui Wang, Liying Yuan, Yanpei Chen, Tianyin Qu, Dong Ou, Wenzhi Shen, Shuang Yang

**Affiliations:** 1 The Third Affiliated Hospital of Zunyi Medical University/First People’s Hospital of Zunyi, Zunyi, 563200 China; 2 Tianjin Key Laboratory of Tumor Microenvironment and Neurovascular Regulation, Medical College of Nankai University, Tianjin, 300071 China; 3 Department of Pathology and Institute of Precision Medicine, Jining Medical University, Jining, 272067 China; 4 Zunyi Medical College, Zunyi, 563006 China; 5 Department of Radiation Oncology, Quanzhou First Hospital Affiliated to Fujian Medical University, Quanzhou, 362000 China; 6 Department of Biochemical Engineering, School of Chemical Engineering & Technology, Tianjin University, Tianjin, 300072 China

**Keywords:** Lung cancer, Molecular biology

**Correction to: Oncogenesis**

10.1038/s41389-019-0162-y published online 24 September 2019

The wrong western blot data was used in Fig. 2d and Fig. 3d in this Article. The correct versions of the figures are provided below. The scientific conclusions of this paper were not affected.

**Figure:**
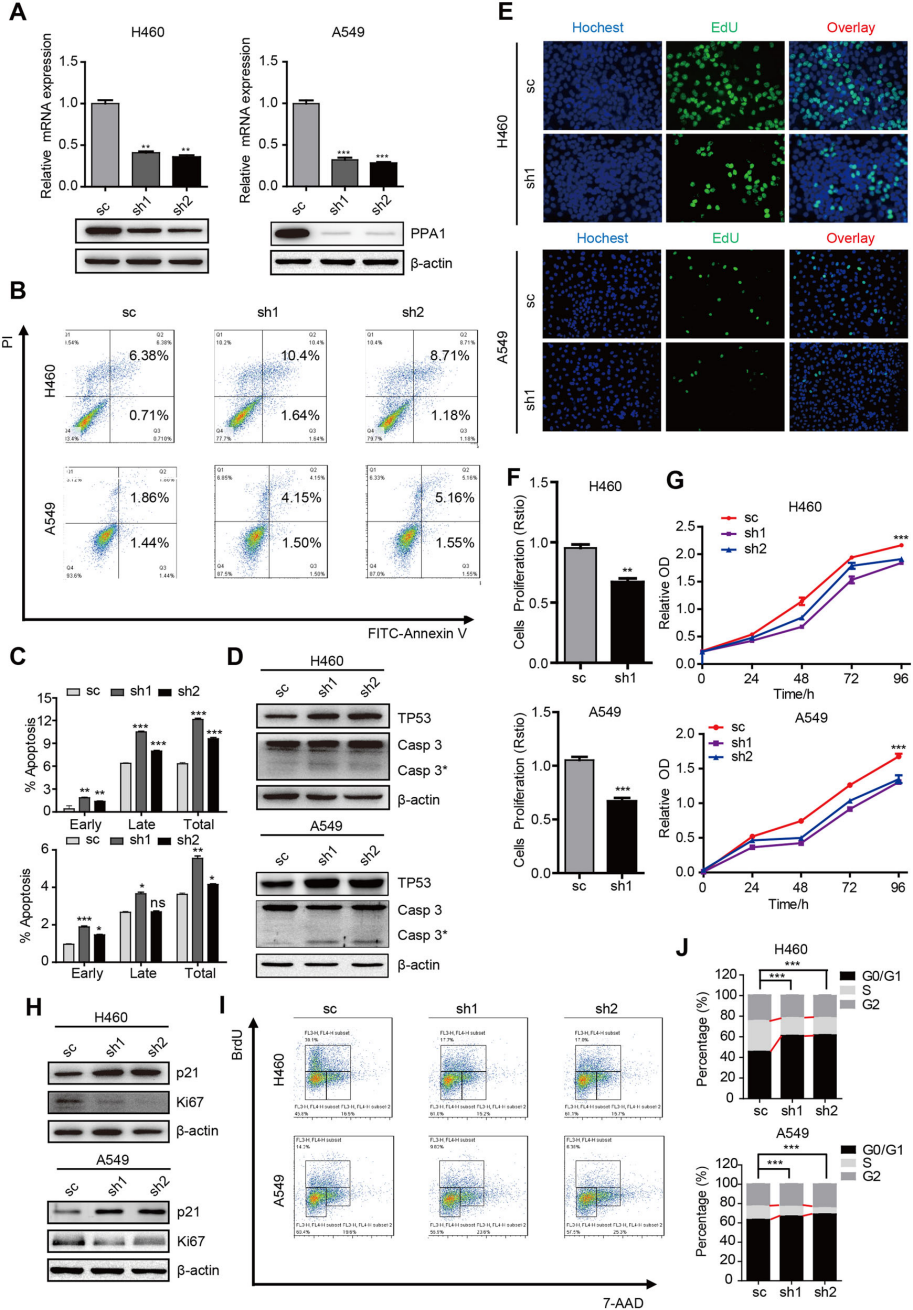
Fig. 2

**Figure:**
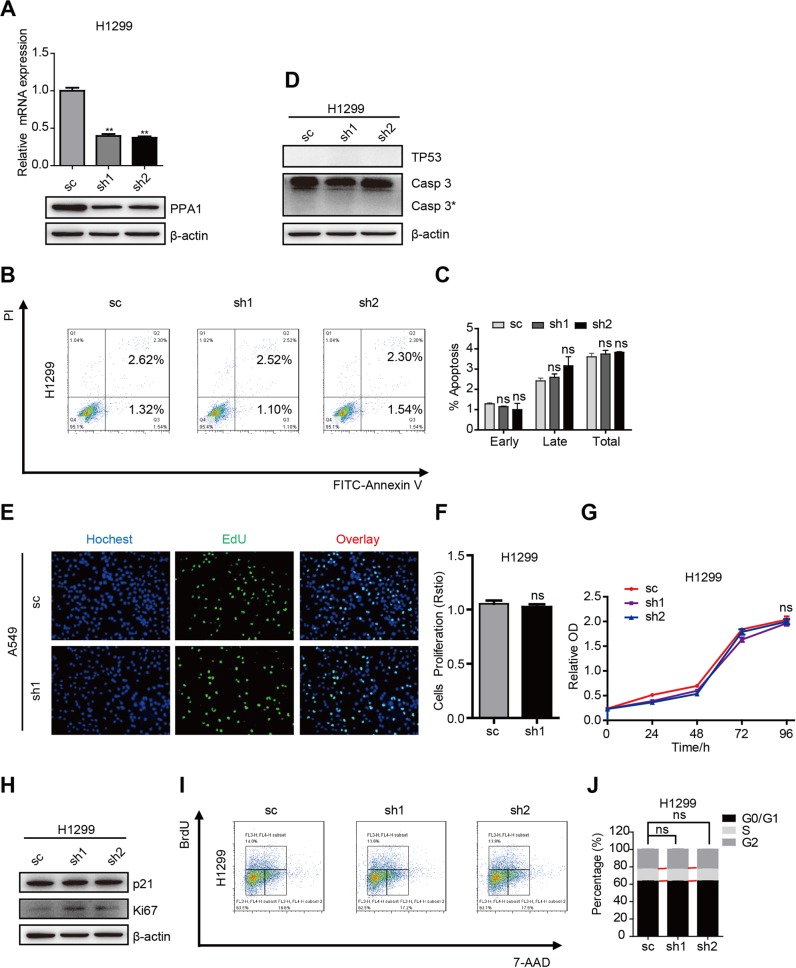
Fig. 3

