# Supplementary material for: PPA1 promotes NSCLC progression via a JNK- and TP53-dependent manner
Source: Oncogenesis. 2019 Sep 24;8(10):53. doi: 10.1038/s41389-019-0162-y (PMC6760234; doi:10.1038/s41389-019-0162-y)
Supplement: Supplementary file 1 — Supplemental tables [file 41389_2019_162_MOESM1_ESM.pdf]

**Supplemental Table 1. Primer sequences**

| Name | Sequence |
|---|---|
| PPA1-sh1 | AAAAGGAATCAGTTGCATGAATATTGGATCCAATATTCATGCAACTGATTCC |
| PPA1-sh2 | AAAAGCTACTGTGGACTGGTTTATTGGATCCAATAAACCAGTCCACAGTAG C |
| SC | AAAAGCTACACTATCGAGCAATTTTGGATCCAAAATTGCTCGATAGTGTAG C |
| PPA1-F | CGGGATCCATGAGCGGCTTCAGCAC |
| PPA1-R | CGACGCGTTTAGTTTTTCTGGTGATGGAAC |
| PPA1-117A-F | ACTGGCTGTTGTGGTGACGCTGACCCAATTGATGTGTGT |
| PPA1-117A -R | ACACACATCAATTGGGTCAGCGTCACCACAACAGCCAGT |
| PPA1-RT-F | CGCTATGTTGCGAATTTGTTC |
| PPA1-RT-R | CCAGTATGTTTATCATTGTGCC |
| GAPDH-RT-F | GGCATCCACGAAACTACCTT |
| GAPDH-RT-R | CTCGTCATACTCCTGCTTGC |
